# A case report of several intraoperative convulsions while using the Narcotrend monitor: Significance and predictive use

**DOI:** 10.1097/MD.0000000000018004

**Published:** 2019-11-22

**Authors:** Joana Berger-Estilita, Katharina Steck, Christian Vetter, Kathleen Seidel, Vladimir Krejci, Darren Hight, Heiko Kaiser

**Affiliations:** a Department of Anaesthesiology and Pain Medicine; b Department of Neurosurgery, Inselspital, Bern University Hospital, University of Bern, Bern, Switzerland.

**Keywords:** brain waves, case reports, depth of anesthesia, epilepsy, intraoperative complications

## Abstract

**Introduction:**

Intraoperative seizures under general anesthesia are rare and our observation is the first to demonstrate a distinct electroencephalogram (EEG) pattern on the Narcotrend monitor.

**Patient concerns:**

We present the case of a 30-year-old man undergoing craniotomy for glioblastoma resection under general anesthesia who suffered tonic-clonic seizures captured in a specific pattern by the intraoperative EEG.

**Diagnoses:**

Our depth of anesthesia monitor recorded, before the seizure, a widening of the beta-wave performance in a distinct “triangular-shaped” pattern. This pattern was repeated before the second seizure. The patient had no previous history of seizures and following surgery no further seizures were recorded.

**Interventions:**

A spectrogram analysis showed a distinct increase in mean absolute beta power immediately prior to the first seizure. The EEG immediately prior to the second seizure was characterized by broadband noise. Both seizures were characterized by increased mean absolute delta, theta, and beta power.

**Outcomes:**

The increase in EEG beta activity seen before the tonic-clonic movements may represent cortical irritability secondary to surgical manipulation, induced by electrical stimulation, reflecting progressive brain over-arousal. The attentive analysis of the relative beta power may have helped forecast the occurrence of the second seizure.

**Lessons:**

We report the use of a simple, inexpensive, and portable EEG-based monitoring device to assist seizure detection and decision making.

## Abstract 2

Supplemental Digital Content is available in the text

## 1 Introduction

Intraoperative seizures (IOS) are a rare, heterogenous event,^[1]^ even in the presence of precipitating factors such as electrical stimulation, direct surgical manipulation, or history of epilepsy.^[2]^ The risk of developing seizures under general anesthesia (GA) is significantly reduced because common hypnotics suppress neuronal activity.

Narcotrend (MonitorTechnik, Bad Bramstedt, Germany) is an electroencephalogram (EEG) monitor designed to measure the depth of anesthesia/hypnosis (DoA).^[3]^ It indicates an individual substage of DoA and displays raw and quantitative EEG data. This device has been used successfully to detect epileptiform patterns in patients anesthetized with volatiles.^[4]^

There are no reports of clinical observation of intraoperative seizures with a motor component and simultaneous recordings of Narcotrend data under total intravenous anesthesia (TIVA).

We present a case of 3 intraoperative generalized tonic-clonic seizures under GA, captured with the Narcotrend in a 30-year-old man undergoing craniotomy for resection of glioblastoma. We describe, for the first time, similar changes in EEG frequency spectra prior to 2 intraoperative seizures, and we hypothesize that this may be predictive of seizures.

## 2 Case report

A 30-year-old man (60 kg, 159 cm) presented for resection of a glioblastoma multiforme (World Health Organization [WHO] grade IV). There were no pre-existing comorbidities, no allergies, and no history of medication or drugs. The glioblastoma was diagnosed incidentally after a sports accident. The patient had undergone a first uneventful craniotomy 3 weeks earlier for tumor biopsy. There had been no preoperative complaints such as headache, paresiae, dizziness, or seizures. All preoperative laboratory data were normal.

The patient did not receive any preoperative anxiolysis or anti-epileptic drugs, according to our internal standards.

On the day of surgery, we established vascular access and monitored the patient according to American Society of Anesthesiology (ASA) standards in the induction suite. Induction of anesthesia with TCI propofol (Schnider) and remifentanyl (Minto) was smooth, and intubation was uneventful. We placed an arterial line in the left radial artery and positioned 3 electrocardiogram electrodes on the patient's forehead (Fpz, F9, and F10 positions) for DoA monitoring. The electrode impedances were below 6000 Ω.

For intraoperative neurophysiological monitoring and mapping, the ISIS system (Inomed Co., Teningen, Germany) equipped with a constant current stimulator used. Motor evoked potentials (MEP) were elicited via transcranial electrical stimulation and, after dura opening, with a strip electrode placed on the precentral gyrus. Details about our neurophysiological set-up have been published elsewhere.^[5,6]^

Ninety minutes after induction, remifentanil was increased to a target blood concentration of 10 μg/mL to provide stable hemodynamics during Mayfield frame application. Propofol target concentration was adapted to show the characteristic frontal EEG signs of unconsciousness with delta (0.1–4 Hz) oscillations and alpha oscillations (8–12 Hz).^[7]^ No local anesthetics were used for incision.

**Figure 1:**
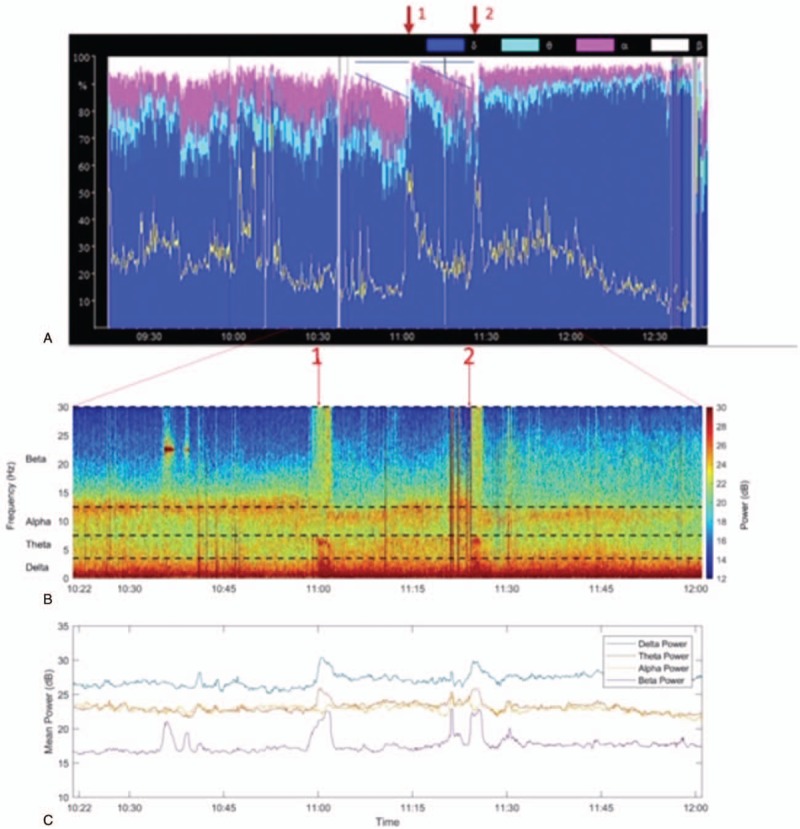
Legend: Evolution of the seizures. A shows the density spectral array (as depicted by a Narcotrend monitor). B shows the spectral relative power (in %) over time. C shows the mean power of main electroencephalographic (EEG) frequencies (analyzed with Matlab). The red arrows in A mark the occurrence of the first and second seizure, respectively. Before each seizure there is a progressive increase of relative power in the beta frequency band, indicated by the triangular shape pattern (A). Before both seizures there is a progressive increase of power in the beta-wave range (C). Before the second seizure the slope/increase of beta power is reduced under higher levels of propofol and dexmedetomidine.

During the dura opening, the patient suddenly suffered tonic-clonic seizures with a motor component, captured by intraoperative EEG (Fig. 1, arrow 1) and electromyography (EMG) (not shown). We immediately increased the propofol-remifentanil TCI infusion to induction dosage. Once the seizure stopped, we administered levetiracetam 1000 mg i.v. to increase the convulsive threshold. Our EEG monitor displayed an increase in relative beta power shortly before the seizure (Fig. 1) and we hypothesized that the plane of anesthesia was too superficial.

Thirty minutes after, the patient developed a second episode of IOS (see Video, Supplemental Video [The video shows the raw EEG signal, captured by the Narcotrend, just before and during the second seizure], showing the raw EEG signal, captured by the Narcotrend, just before and during the second seizure and Fig. 1, arrow 2). We immediately increased the propofol-remifentanil TCI infusion and administered thiopental until the EEG displayed burst suppression. We then started dexmedetomidine 2 μg/kg/h as a continuous infusion. In retrospect, we could see a pattern of beta power increase shortly before the second seizure in the Narcotrend again (Fig. 1A).

Ninety minutes after the first seizure, a third episode of IOS clinically manifested under a burst suppression EEG (not shown). A bolus dose of propofol was used to end the seizure, which stopped after 30 seconds. We added phenytoin 750 mg i.v. as anticonvulsant and repeated levetiracetam 1 g i.v. The complete duration of surgery was 7 hours and 45 minutes and no further seizures occurred.

Afterwards, the patient was transferred to an intensive care unit and extubated uneventfully overnight. He showed a left-sided hemiplegia immediately after surgery which improved in the course of the following days. No more seizures were reported. One year after surgery, he is no longer on anti-epileptic drugs, had no further convulsions, and has returned to work. He has only fine motor impairment of the left hand.

The relative spectral power display from our EEG monitor showed a progressive increase in relative power in the beta-wave range, accompanied by a reduction in relative power in the delta-wave range preceding the first 2 seizures (Fig. 1A). This occurred 8 to 10 minutes before the motor component. To enable more finely grained spectral observations, we also created a spectrogram using Matlab (Matlab R2017b, The Mathworks, Natick, MA) with a moving window of 2 seconds (1 second overlap) (Fig. 1C). There was a distinct increase in mean absolute beta power immediately prior to the first seizure, but not in the alpha, theta, and delta bands. The EEG immediately prior to the second seizure was characterized by broadband noise. Both seizures were characterized by increased mean absolute delta, theta, and beta power. The slow increase in relative beta power prior to the seizures appears to be associated with a broadening alpha oscillation.

The Bern Cantonal Ethics Committee (Kantonale Ethikkomission [KEK] Bern) waived the need for ethics approval (Req-2018-00985) because the case report does not fall into the Swiss Law for Human Research (Art.2) and the patient gave written informed consent for publication.

## 3 Discussion

The functional relevance of brain oscillations is still partly unknown, but a well-established fact is that different frequencies reflect global state changes of the brain. Fast rhythms can indicate enhanced arousal and can be distinguished by specific interaction patterns. Beta oscillations are observed frequently in states of vigilance and paradoxical excitation after propofol administration,^[8]^ suggesting a more superficial plane of anesthesia. They are, however, a rare seizure pattern.^[9]^

We hypothesize that the increase in beta activity in this patient seen before the tonic-clonic movements may represent cortical irritability secondary to surgical manipulation, induced by electrical stimulation. This is based on a pre-ictal rise in EEG activity and a steady increase of beta activity in the EEG. Such an increase may be secondary to a frequency shift of thalamocortical oscillations towards higher frequencies before the convulsion, representing a rise in glutaminergic activity^[8]^ and progressive brain over-arousal.

Importantly, we believe that attentive analysis of the Narcotrend relative beta power may have helped forecast the occurrence of the second seizure. The transition from an “anesthetised” to an “epileptic” state, repeated in the 2 observations, supports the triggering of a seizure by purposefully inducing a change between brain states in the presence of appropriate stimulation.^[10]^ Recent computational models of brain state transitions have successfully used a predictability algorithm based on EEG beta power analysis.^[11]^ Such models are, however, still in an experimental phase, and are time-consuming and expensive. They also rely on retrospective data analysis, which is not suitable for use “at the bedside.” The Narcotrend, which is a relatively inexpensive, portable device able to transmit and analyse EEG data in real time, could potentially overcome these drawbacks.

There are several limitations to our discussion. First of all, it is not known to what degree an increase in beta power might be due to increased muscle activity or electrical interference, as beta waves can be associated with muscle contractions elicited by electrical stimulation.^[12]^

Additionally, we have been unable to reproduce or generalize these findings. Depth of anesthesia monitors are primarily designed to estimate the level of anesthesia, and not for diagnosis or prediction of intraoperative epileptic activity, so we are reporting an “off-label” use. Since this is a case report, the signal that was picked up, may have occurred coincidentally, although we find it intriguing that it occurred twice.

Regarding patient safety, it is known that intraoperative stimulation for motor evoked potential monitoring might be associated with concern of inducing IOS. The reported incidence ranges from 0.7% to 4.4%,^[2,13]^ but it is commonly accepted that electrical brain stimulation can be performed in patients with symptomatic seizures. In a series of >4000 monitored patients, most of the patients with an IOS experienced only 1 (78%) or 2 (16%) seizures, but multiple IOS were rare (6.2%).^[2]^ The simplified 3 to 4-channel EEG and the free-running EMG aids in detecting IOS early, but the information from the DoA monitor could have helped anticipate further IOS.

Finally, there are other competitor devices also have the capacity to provide raw EEG signals capable of upload and analysis. However, the Narcotrend monitor has unique advantages^[3]^: it provides a vast amount of information and the raw EEG signal can be recorded by standard electrocardiogram electrodes. That being said, we cannot safeguard that this electroencephalography brain monitor is better at assisting seizure detection than others available.

In conclusion, we describe a previously unreported strategy of IOS suspicion involving observation of a characteristic EEG pattern on a DoA monitor. The strength of this case report lies in the demonstration of the possibility of use of simple EEG-based monitoring to assist seizure detection and decision making, giving encouragement to the development of better, more-purpose built, devices in the future.

## Acknowledgments

This manuscript adheres to the applicable CARE guidelines. The authors thank Prof. Dr. Andreas Raabe for his outstanding contribution to the case. Prof. Raabe gave permission to be named.

## Author contributions

**Conceptualization:** Joana M. Berger, Vladimir Krejci.

**Data curation:** Katharina Steck, Christian Vetter, Heiko Kaiser.

**Formal analysis:** Kathleen Seidel, Darren Hight, Heiko Kaiser.

**Methodology:** Christian Vetter, Darren Hight.

**Software:** Vladimir Krejci.

**Supervision:** Heiko Kaiser.

**Visualization:** Vladimir Krejci.

**Writing – original draft:** Joana M. Berger, Katharina Steck.

**Writing – review & editing:** Christian Vetter, Kathleen Seidel, Vladimir Krejci, Darren Hight, Heiko Kaiser.

## Supplementary Material

Supplemental Digital Content
